# Spatiotemporal Variability in and Factors Associated with Wet Deposition of Atmospheric Mercury in China from 2000 to 2022

**DOI:** 10.3390/toxics14100879

**Published:** 2026-09-30

**Authors:** Xi Cheng, Zhihao Zeng, Jiaqi Wu, Chenxi Zhao, Shuhua Zeng, Lei Liu

**Affiliations:** 1 School of Chemical and Environmental Engineering, China University of Mining and Technology—Beijing, Beijing 100083, China; 2310340229@student.cumtb.edu.cn (Z.Z.); 2310340225@student.cumtb.edu.cn (J.W.); 2310340311@student.cumtb.edu.cn (C.Z.); 2310340310@student.cumtb.edu.cn (S.Z.); 2 State Key Laboratory of Severe Weather Meteorological Science and Technology, Chinese Academy of Meteorological Sciences, Beijing 100081, China; 3 Key Laboratory of Atmospheric Chemistry of CMA, Chinese Academy of Meteorological Sciences, Beijing 100081, China

**Keywords:** mercury, wet deposition, precipitation chemistry, random forest, SHAP, China

## Abstract

The wet deposition of atmospheric mercury (Hg) transfers Hg to terrestrial and aquatic ecosystems, yet China lacks spatially consistent, long-term monitoring, a critical gap given its high and spatially heterogeneous anthropogenic Hg emissions. We compiled candidate observations from 58 published studies at 120 coordinate sites and used a random forest model to reconstruct the wet Hg deposition from 2000 to 2022. Site-grouped five-fold validation gave an overall coefficient of determination (*R*^2^) of 0.596 and root mean square error (RMSE) of 47.80 µg m^−2^ yr^−1^; leave-one-region-out validation was weaker (RMSE = 66.29 µg m^−2^ yr^−1^). The reconstructed national annual mean flux ranged from 20.06 to 26.19 µg m^−2^ yr^−1^, and the derived volume-weighted mean (VWM) Hg concentration in precipitation ranged from 28.02 to 37.12 ng L^−1^. The concentration estimate was highest in 2022, while flux remained strongly associated with rainfall. The high-rainfall southern regions had lower VWM concentrations and high fluxes; the drier northwestern regions had relatively high concentrations but low fluxes. Transport sector value-added, coal consumption, industrial value-added, and precipitation ranked highest in Hg input importance, as characterized by the SHapley Additive exPlanations (SHAP) method, where the socioeconomic variables were correlated proxies rather than direct measures of Hg emissions. The joint interpretation of concentration, flux, and precipitation is essential for assessing ecosystem Hg loading, although regional transfer and local extremes remain uncertain.

## 1. Introduction

Mercury (Hg) is a persistent, globally transported toxic metal that cycles among the atmosphere, terrestrial surfaces, and aquatic ecosystems. Atmospheric oxidation, particle association, wet and dry deposition, and surface re-emission redistribute Hg far from primary sources and deliver it to soils, waters, wetlands, reservoirs, and food webs [1,2,3,4]. Wet deposition is an important transfer pathway because precipitation removes soluble oxidized Hg and particle-bound Hg from the atmosphere [5,6]. Its magnitude depends on atmospheric Hg loading and speciation as well as precipitation amount, storm frequency, and in-cloud and below-cloud scavenging [3,5,6,7]. Photochemical and aqueous-phase reactions alter the abundance of scavengeable Hg species [8,9], while deposited Hg may be retained, transported, methylated, or re-emitted [10,11]. The Hg concentration in precipitation and wet-deposition flux are therefore related but distinct indicators: concentration describes Hg in rain or snow, whereas flux integrates that concentration with precipitation amount and represents mass loading to ecosystems. Subsequent retention, methylation, and food web transfer affect ecological and human exposure; biomonitoring addresses uptake rather than deposited mass [12,13].

China is a critical region for evaluating atmospheric Hg deposition because of its historically large Hg production, use, and anthropogenic emissions [14,15]. Emission inventories have identified coal combustion, nonferrous metal smelting, cement production, industrial boilers, and waste-related activities as major sources of speciated atmospheric Hg [16,17,18,19,20,21,22,23]. These inventories describe rapid Hg emission growth during industrialization, an emission peak around 2010–2011, and subsequent reductions associated with multipollutant controls, ultra-low-emission retrofits, and source-specific mitigation [17,18,19,20,21,22]. Long-term observations at Chinese background sites likewise show declining gaseous elemental Hg levels after strengthened air-pollution controls [24,25]. Wet deposition, however, does not necessarily decline in direct proportion to total emissions because it also depends on emitted Hg species, regional transport, source composition, and precipitation variability. The deposition efficiency of cement emissions, for example, can be relatively high because they contain a substantial soluble divalent Hg fraction [26], while trade and source-apportionment studies demonstrate that production, consumption, and atmospheric transport redistribute Hg deposition [27,28].

Long-term, spatially continuous monitoring of wet Hg deposition remains sparse in China. Published measurements cover mining districts, urban industrial centers, coastal and island sites, reservoirs, wetlands, agricultural watersheds, background regions, and southwestern biodiversity hotspots, revealing pronounced contrasts among source environments and precipitation regimes [12,29,30,31,32,33,34,35,36,37,38,39,40,41]. Complementary evidence from continuous atmospheric monitoring in Xi’an [42], dispersion and lichen surveys near a former chlor-alkali plant [43], and moss biomonitoring in a legacy mining district [13] shows that air concentrations, transport patterns, and biological accumulation capture different components of Hg exposure; none provides a spatially continuous, multi-decadal record of wet deposition flux across China. Linking measured air quality with blood heavy-metal concentrations further demonstrates the complementary roles of environmental monitoring and biomonitoring in assessing potential human exposure [44]. Moreover, previous wet deposition records differ in sampling duration, site type, analytical method, and spatial representativeness, and both observations and simulations contain substantial uncertainty [45]. An urgent task is to assess the broad-scale temporal and regional patterns that can be inferred from these previous dispersed observations. We therefore compiled Chinese wet Hg deposition observations from 2000 to 2022 and combined them with socioeconomic, meteorological, and land surface predictors in a random forest model. We examined whether this heterogeneous evidence base could support a stable broad-scale reconstruction, whether precipitation Hg concentration and deposition flux followed different temporal and regional trajectories, and which predictors were most strongly associated with the modeled flux. We expected emission control signals to be more apparent in concentration than in flux because interannual precipitation variability directly modulates mass deposition based on the related associations and interpretations of the model.

## 2. Materials and Methods

### 2.1. Wet Deposition Data Compilation

Published measurements of wet deposition of atmospheric Hg in China were compiled for model development and evaluation. Web of Science (WoS) and China National Knowledge Infrastructure (CNKI) were searched for studies containing sampling periods beginning in 2000–2022, irrespective of publication year. The final search was conducted on 28 August 2025. A broad search strategy incorporating Hg and other trace metal terms was used to capture studies in which wet Hg deposition was reported together with other measured analytes.

The following keyword combination was used: TOPIC: (“trace element” OR “toxic element” OR “heavy metal” OR arsenic OR cadmium OR chromium OR copper OR mercury OR nickel OR lead OR zinc) AND TOPIC: (atmospheric NOT soil) AND TOPIC: (deposition OR dustfall OR dust OR “bulk deposition”) NOT TOPIC: (moss). Studies were subsequently restricted to those conducted within China during the screening process. The searches identified 416 records from WoS and 442 from CNKI (858 records in total). After removal of six duplicate records across the two databases, 852 unique records remained. Initial relevance screening excluded 673 records, leaving 179 records for further assessment. A total of 58 studies met the eligibility criteria and contributed 148 candidate observations. Studies were eligible when the site was in China; location and sampling period were identifiable; wet Hg deposition flux was reported or calculable from precipitation’s total Hg concentration and depth measured over the same interval; and units, duration, and source information permitted harmonization. Records were excluded when these conditions were not met, including reports of dry or bulk deposition without separable wet deposition values. The final compiled dataset is provided in Data S1 in the Supplementary Materials.

When concentration *C* (ng L^−1^) and precipitation depth *P* (mm) covered the same interval, wet deposition flux was calculated as *C* × *P*/1000 (µg m^−2^ for that interval). Fluxes were harmonized to µg m^−2^ yr^−1^, where yr denotes one year. Records with ambiguous units, incompatible periods, or insufficient sampling information were excluded. An interval that began in 2022 and ended in 2023 was retained; the national reconstruction was restricted to 2000–2022.

### 2.2. Predictors and Data Preprocessing

Predictors represented three process domains relevant to wet Hg deposition: anthropogenic activity and emission background, meteorological scavenging, and land surface exchange. Because sector-resolved Hg emission inventories were not available for every year at a consistent spatial scale, socioeconomic indicators were used as proxies for emission intensity and human activity. This approach follows spatial environmental models that use activity, land use, and meteorological variables when direct emissions are incomplete [46,47]. The 11 predictors were coal consumption, cement production, steel production, industrial value-added, transport sector value-added, precipitation, air temperature, relative humidity, wind speed, solar radiation, and cropland area. Coal consumption represented combustion-related emissions; cement and steel production represented high-temperature industrial activity; and industrial and transport value-added represented broader urban economic activity rather than source-specific Hg emission inventories. Precipitation represented wet scavenging capacity, while temperature, relative humidity, wind speed, and solar radiation characterized conditions related to transformation, transport, and removal. Cropland area was included as a land surface and human activity proxy.

Annual predictor records were assembled and averaged for 2000–2022 and matched to each observation in the literature by sampling period and the most precise reported location. Meteorological variables came from the China Meteorological Forcing Dataset version 2.0 (CMFD 2.0; 0.1° grid, 3-hourly native resolution) [48]. Precipitation was aggregated as depth; temperature, relative humidity, wind speed, and solar radiation were summarized over the corresponding interval. The Hg observations were not spatially interpolated: gridded predictor values were extracted at reported coordinates or summarized for the reported administrative unit. Socioeconomic variables were compiled from provincial annual statistics and assigned by province and year. A cropland indicator came from the supplied 30 m GLC_FCS30-family land cover product [49], aggregated to the modeling grid; the supplied national values were not interpreted as verified area fractions. Predictor sources and native resolutions are listed in Table S1 in the Supplementary Materials.

Socioeconomic and land surface variables identified as strongly right-skewed were log-transformed, and continuous predictors were standardized within each training fold before modeling. The response variable, wet Hg deposition flux, remained on its original scale to preserve physical interpretation and comparison with reported observations. Sensitivity tests considered records close to one year in duration and excluded the shortest records. Predictions were interpreted at broad regional and multi-year scales rather than as event- or site-specific estimates.

### 2.3. Model Development and Evaluation

A random forest regression model was used to estimate the wet Hg deposition flux. Random forest combines bootstrap resampling with random selection of candidate predictors at each split, reducing variance relative to a single decision tree [50]. The model was implemented in Python 3.13.3 with scikit-learn [51]. For regression, the final prediction is the mean of the predictions from all trees:

$$\hat{y}=\frac{1}{N_{\mathrm{trees}}}\sum_{i=1}^{N_{\mathrm{trees}}}T_{i}\left(x\right)$$

where $\hat{y}$ is the model prediction, *N_trees_* is the number of decision trees, and *T_i_*(*x*) is the prediction of the *i*-th decision tree for input vector *x*.

For the primary evaluation, observations were partitioned into five folds by coordinate site, so records from a site could not appear in both training and test subsets. To limit overfitting in the small and heterogeneous dataset, the model used 500 trees, a maximum depth of 5, at least 3 samples per leaf, at least 6 samples per split, and max_features = $\sqrt{p}$, where *p* is the number of predictors. The same data were also evaluated with flux-quartile-stratified site-grouped folds, leave-one-region-out validation, and 50 repeated random 3:1 splits [52,53]. The primary performance estimates were calculated from the predictions made when each site was held out in one of five folds. Leave-one-region-out validation tested transfer to each of the seven regions; the 50 repeated random splits provided a less independent reference. Site grouping prevented repeated-site leakage but did not fully separate neighboring sites or observations drawn from the same study.

Model performance was quantified using the coefficient of determination (*R*^2^) and root mean square error (RMSE):

$$R^{2}=1-\frac{\sum_{i=1}^{n}(y_{i}-{\hat{y}}_{i}{)}^{2}}{\sum_{i=1}^{n}(y_{i}-\bar{y}{)}^{2}}$$

$$\mathrm{RMSE}=\sqrt{\frac{1}{n}\sum_{i=1}^{n}(y_{i}-{\hat{y}}_{i}{)}^{2}}$$

where *y_i_* is the observed value, ${\hat{y}}_{i}$ is the predicted value, $\bar{y}$ is the mean observed value, and *n* is the number of samples.

National annual wet deposition fluxes were summarized using cosine-of-latitude area weighting. Total Hg concentration in precipitation was derived from predicted flux and gridded precipitation as precipitation volume-weighted mean (VWM), consistent with wet deposition network practice [6]. SHapley Additive exPlanations (SHAP) was used to decompose the predictions from the model fitted to the observations [54]. Mean absolute SHAP values were calculated across all samples and normalized to sum to 100% to rank predictors within this fitted model. SHAP summary plots show the distribution of observation-level associations but do not establish independent causal effects.

## 3. Results and Discussion

### 3.1. Model Performance

The observations used for modeling were strongly right-skewed. Their median was 17.10 µg m^−2^ yr^−1^, with an interquartile range of 6.43–34.85 µg m^−2^ yr^−1^; 113 values were at or below 50 µg m^−2^ yr^−1^, and eight exceeded 200 µg m^−2^ yr^−1^. The 120 coordinate sites span 83.137–128.155° E and 21.612–47.177° N. Legacy mining districts can receive exceptionally high deposition from local Hg-enriched sources and contaminated surfaces [29]. Urban and industrial sites in Changchun, Chongqing, Nanjing, and Qingdao reflect the varying effects of combustion, industrial activity, particulate pollution, and rainfall scavenging [12,30,31,32,33,34,35,36]. Rural, background, coastal, wetland, watershed, and mountain sites instead integrate different combinations of regional transport, surface exchange, and precipitation regime [37,38,39,40,41]. The compiled observations should therefore be treated as a heterogeneous literature sample, not as a uniformly designed national monitoring network.

**Figure 1:**
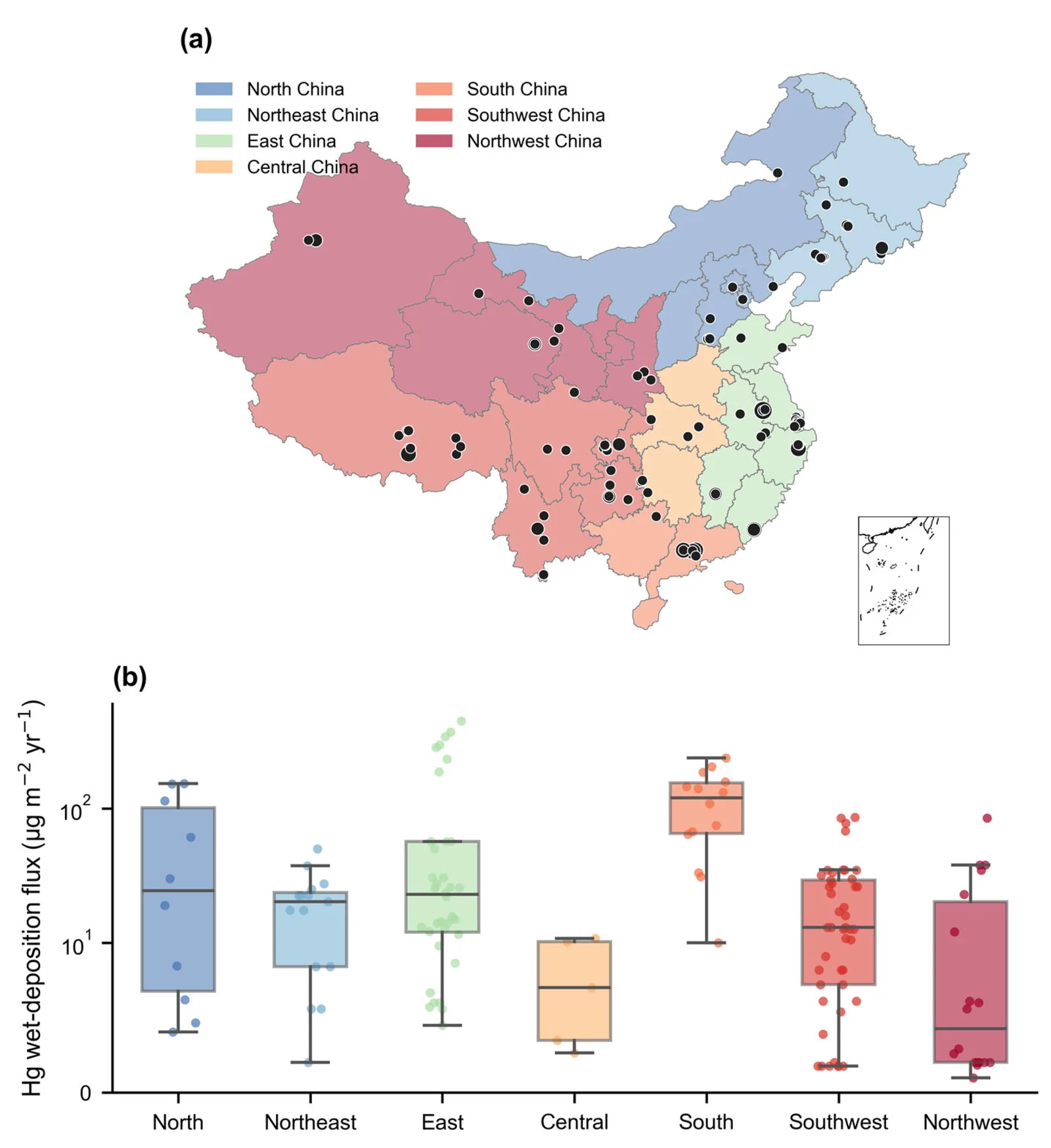
Spatial coverage and regional distributions of the wet Hg deposition observations. (**a**) The 120 coordinate sites and seven analysis regions. (**b**) Points show observations; boxes show medians and interquartile ranges, and whiskers extend to 1.5 times the interquartile range. These summaries are descriptive; no significance test was applied to differences among regions.

Regional distributions differed in both central tendency and dispersion (Figure 1). The largest value was observed in East China, 447.68 µg m^−2^ yr^−1^, reported for a Shanghai functional zone [55]. South China had the highest observation median (119.84 µg m^−2^ yr^−1^), whereas Northwest China had the lowest (4.26 µg m^−2^ yr^−1^). This value represents a site-specific urban or coastal setting and should not be generalized to the region. South China had a comparatively high median but fewer extreme values, consistent with abundant rainfall increasing mass deposition while diluting the Hg concentrations in precipitation [12,35]. The Chongqing observations show that emissions, particulate matter, rainfall, and methylmercury inputs can interact in urban wet deposition [31,32,33], while Qingdao and Chongming studies demonstrate the additional effects of coastal transport and seasonal rainfall [36,37,38]. In southwestern China, sustained high rainfall can also produce ecologically relevant deposition in areas where local emissions are not dominant [41].

These sampling characteristics constrain the attainable resolution of the statistics. Annual socioeconomic and aggregated meteorological predictors can represent broad gradients but not near-source plumes, short sampling periods, unusual rainfall sequences, sampler differences, or legacy contamination. Accordingly, statistical evaluation focused on the central observation range and regional contrasts; rare high-flux observations were retained as evidence of local conditions but were not expected to be reproduced accurately by the national reconstruction.

**Figure 2:**
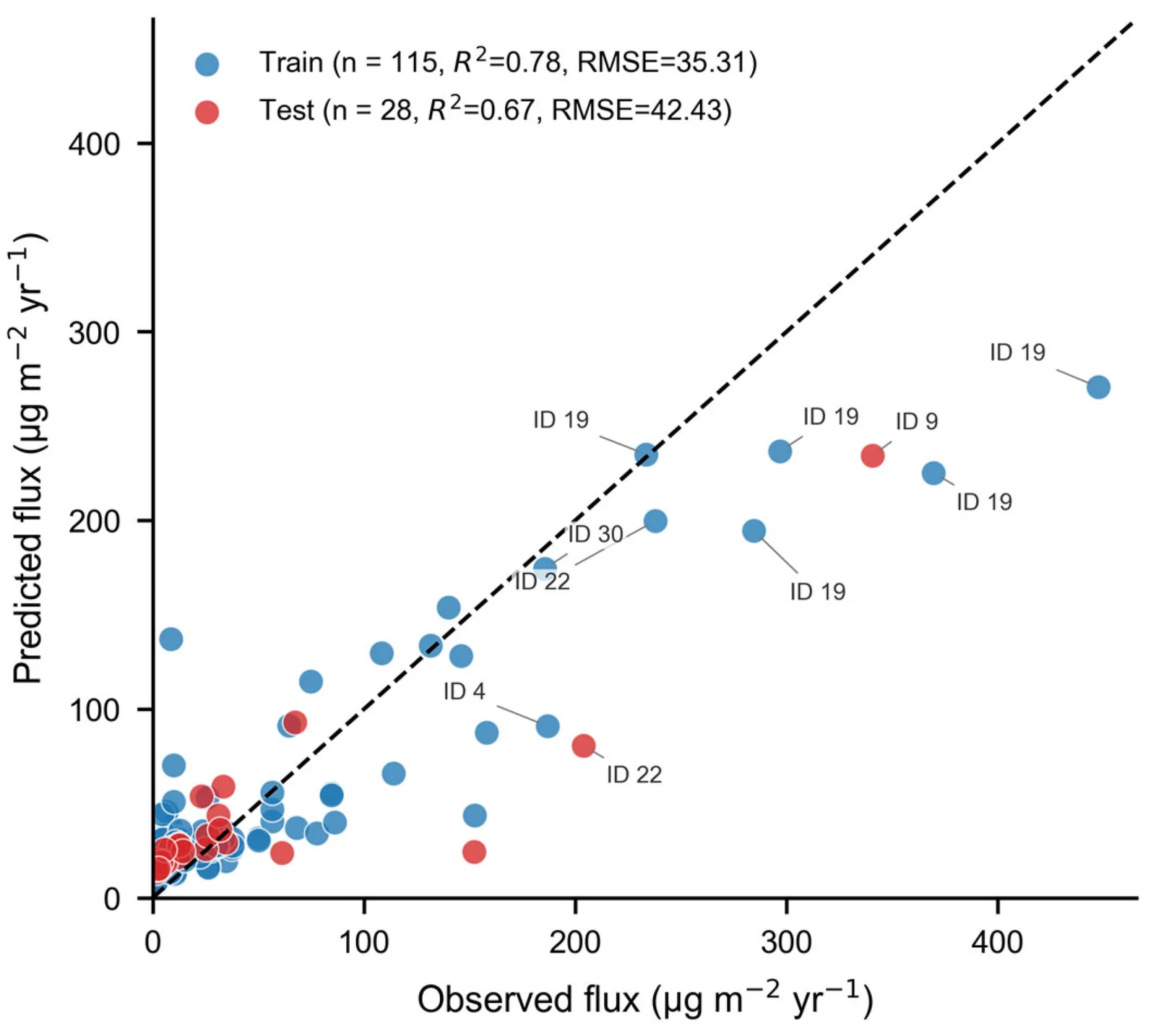
Observed versus random forest estimates of wet deposition flux of Hg for the third site-grouped fold. Of the 148 candidate observations, 143 had complete predictor inputs for model evaluation, comprising 115 training and 28 held-out test observations. Each point represents one harmonized site-period observation; one study could contribute multiple sites or sampling periods. The 10 observations with the largest measured fluxes are labeled with the literature Source ID. These observations correspond to Source IDs 4, 9, 19, 22, and 30. The complete mapping of all 58 Source IDs is provided in Data S1. The dashed line denotes 1:1 agreement.

Figure 2 shows one site-grouped training/test fold with training and held-out observations. Training performance was *R*^2^ = 0.78 and RMSE = 35.31 µg m^−2^ yr^−1^; test performance was *R*^2^ = 0.67 and RMSE = 42.43 µg m^−2^ yr^−1^. The gap and substantial held-out error limited precise site-level interpretation.

Predictions were systematically compressed toward the center of the distribution and underestimated many observations above approximately 200 µg m^−2^ yr^−1^ (Figure 2). This behavior is consistent with the scarcity of extreme samples and the averaging behavior of tree ensembles [45,46,47,50]. When the two or five highest observations were omitted and the models refitted, the site-grouped RMSE change from 47.8 to 50.1 and 40.1, respectively (Figure S1). These sensitivity subsets had different response distributions, so their RMSE values should not be read as direct gains in predictive skill, considering that the high values therefore contributed materially to *R*^2^.

**Figure 3:**
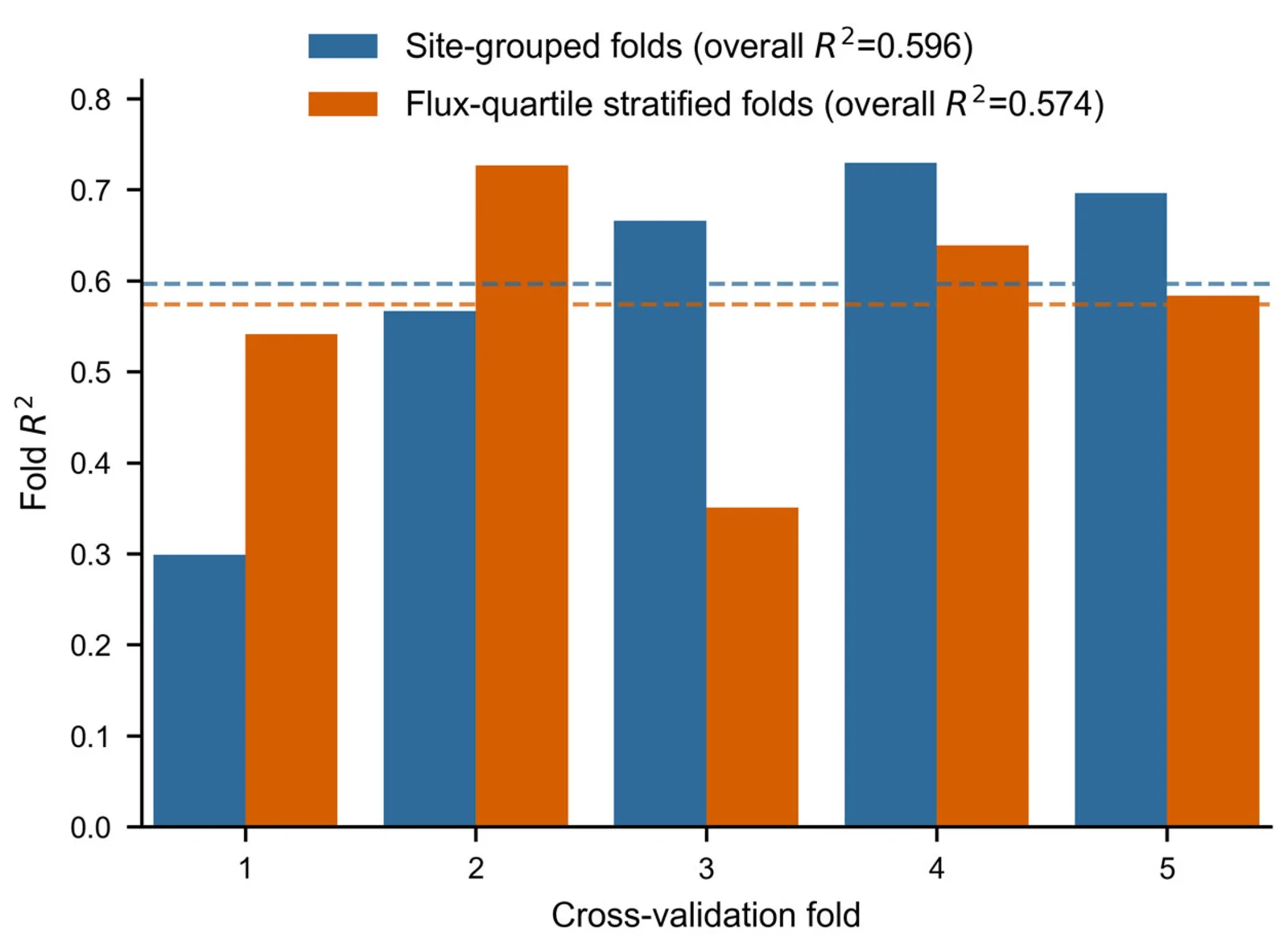
Fold-specific *R*^2^ in five-fold random forest cross-validation. Blue bars: site-grouped primary validation; orange bars: site-grouped sensitivity validation approximately stratified by observed-flux quartile. Dashed lines show *R*^2^ calculated from all out-of-fold predictions in each scheme.

Across the primary five site-grouped folds, the pooled out-of-fold *R*^2^ was 0.596 and RMSE was 47.80 µg m^−2^ yr^−1^. The fold-specific *R*^2^ ranged from 0.299 to 0.730 (Figure 3). The flux-quartile-stratified site-grouped sensitivity split had a pooled *R*^2^ = 0.574 and an RMSE = 49.11 µg m^−2^ yr^−1^. Leave-one-region-out validation was weaker (RMSE = 66.29 µg m^−2^ yr^−1^), showing limited transfer to the entire unsampled regions (Table S2). The fifty random 3:1 split gave a median test *R*^2^ of 0.503 but did not substitute for spatial evaluation. In summary, transfer across regions was substantially weaker, and rare local extremes was poorly reproduced. Our following analyses therefore emphasize broad temporal and regional patterns and model associations rather than exact local values.

### 3.2. Spatiotemporal Patterns of Wet Deposition of Hg

**Figure 4:**
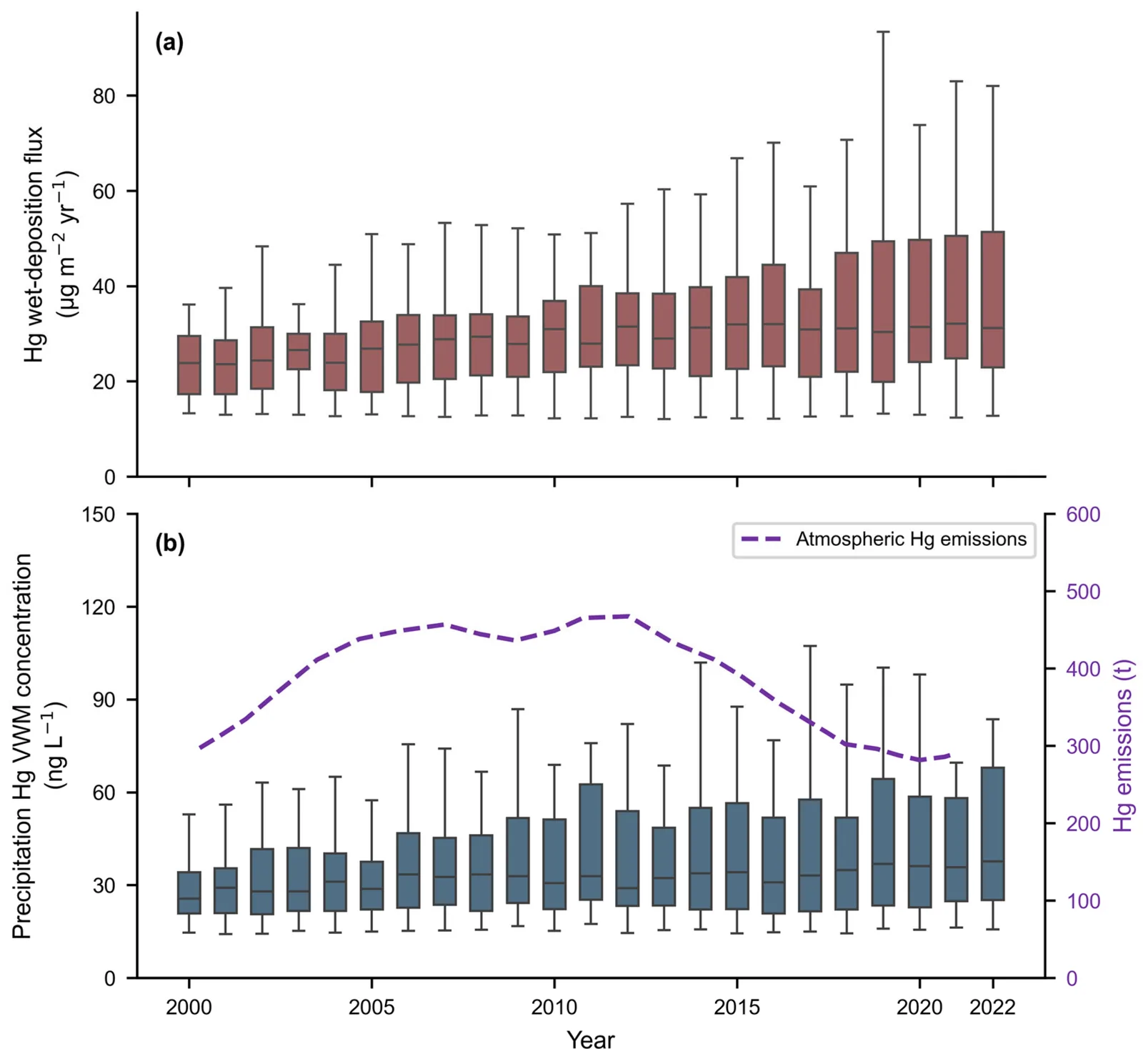
Annual province-level distributions of (**a**) modeled wet deposition flux of Hg and (**b**) derived precipitation Hg volume-weighted mean (VWM) concentration during 2000–2022. Boxes show the interquartile range and median across provinces; whiskers show the wider spatial distribution. The dashed series in panel (**b**) shows reported atmospheric Hg emissions from Ouyang et al. [22] on the right axis.

During 2000–2022, the reconstructed national annual mean wet deposition flux ranged from 20.06 to 26.19 µg m^−2^ yr^−1^, whereas the derived precipitation Hg volume-weighted mean (VWM) concentration ranged from 28.02 to 37.12 ng L^−1^ (Figure 4). Rural and background sites commonly have lower concentrations and fluxes [35,36,37], whereas urban, industrial, mining, reservoir, and watershed studies have reported higher values and stronger event effects [12,29,31,32,33,34,36,39,40,55]. The national ranges reflect both the literature sample and the predictors used for extrapolation.

Flux and concentration followed different trajectories (Figure 4). The national modeled mean flux rose from 20.53 µg m^−2^ yr^−1^ in 2000 to 25.72 µg m^−2^ yr^−1^ in 2022 and reached 26.19 µg m^−2^ yr^−1^ in 2021. The derived VWM concentration was 28.31 ng L^−1^ in 2000 and 37.12 ng L^−1^ in 2022. Thus, the model does not support a sustained decrease in the Hg concentration in precipitation after the early 2010s. Concentration and flux must therefore be interpreted jointly. Precipitation Hg concentration describes the Hg mass per unit precipitation, whereas flux integrates concentration with rainfall amount and removal. Here, the national concentration was derived from modeled flux and gridded precipitation, not measured independently across China (Figure S2). This cannot alone establish an atmospheric response to emission controls. For ecosystem assessment, total deposited mass is the relevant input; subsequent environmental fate and exposure require separate evidence.

The temporal pattern cannot by itself demonstrate an effect of emission controls. The inventories indicate that Chinese anthropogenic atmospheric Hg emissions rose through the 2000s, peaked around 2010–2011, and subsequently declined [16,17,18,19,20,21,22]. Background observations show declining gaseous elemental Hg levels after strengthened air pollution controls [24,25]. In contrast, the derived precipitation Hg concentration in this reconstruction reached its highest value in 2022. The discrepancy may be due to precipitation, source composition, study coverage, or predictor limitations, and these data cannot separate their effects.

**Figure 5:**
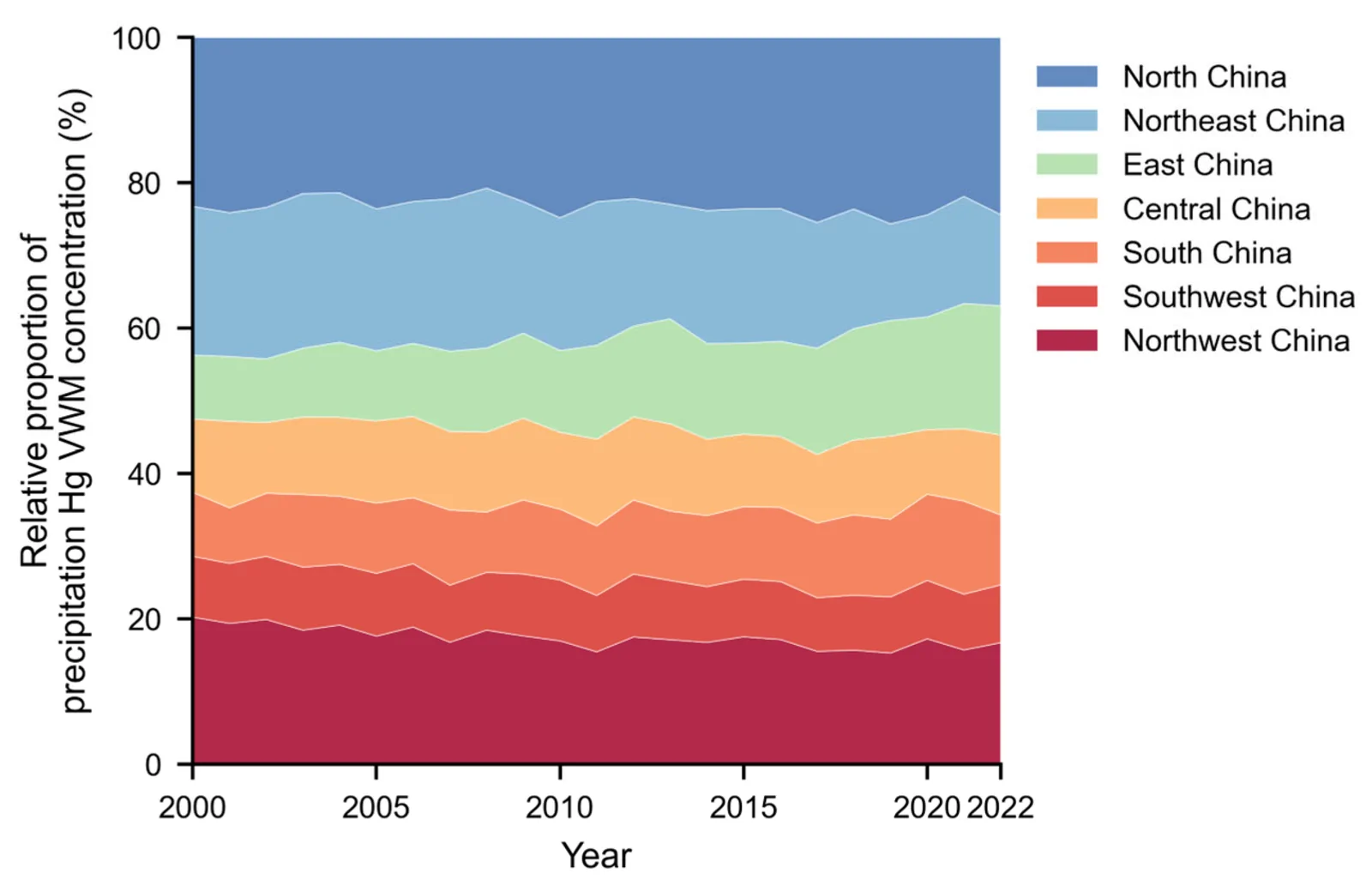
Normalized regional composition of modeled precipitation Hg volume-weighted mean (VWM) concentrations during 2000–2022. Percentages were calculated from regional concentration indices for comparison and must not be interpreted as regional contributions to national Hg mass deposition.

The normalized regional comparison in Figure 5 shows how modeled VWM concentrations were distributed among broad geographic regions. Because concentration is not an additive mass quantity, these percentages should be interpreted as relative regional indices, not as contributions to national Hg deposition. North and East China generally occupied large shares of the normalized concentration signal, consistent with the spatial clustering of coal combustion, industrial boilers, cement production, metallurgy, and consumption-linked deposition [16,17,18,19,20,21,22,23,24,25,26,27,28]. Northwest China’s variable share may reflect the combination of low rainfall, pollutant accumulation, and episodic removal; continuous atmospheric measurements in Xi’an independently document strong anthropogenic influence in this region [42]. Coastal East China was characterized by source intensity, regional transport, and effective wet removal, whereas southern and southwestern high-rainfall regions were more strongly shaped by precipitation and ecological sensitivity [12,41].

**Figure 6:**
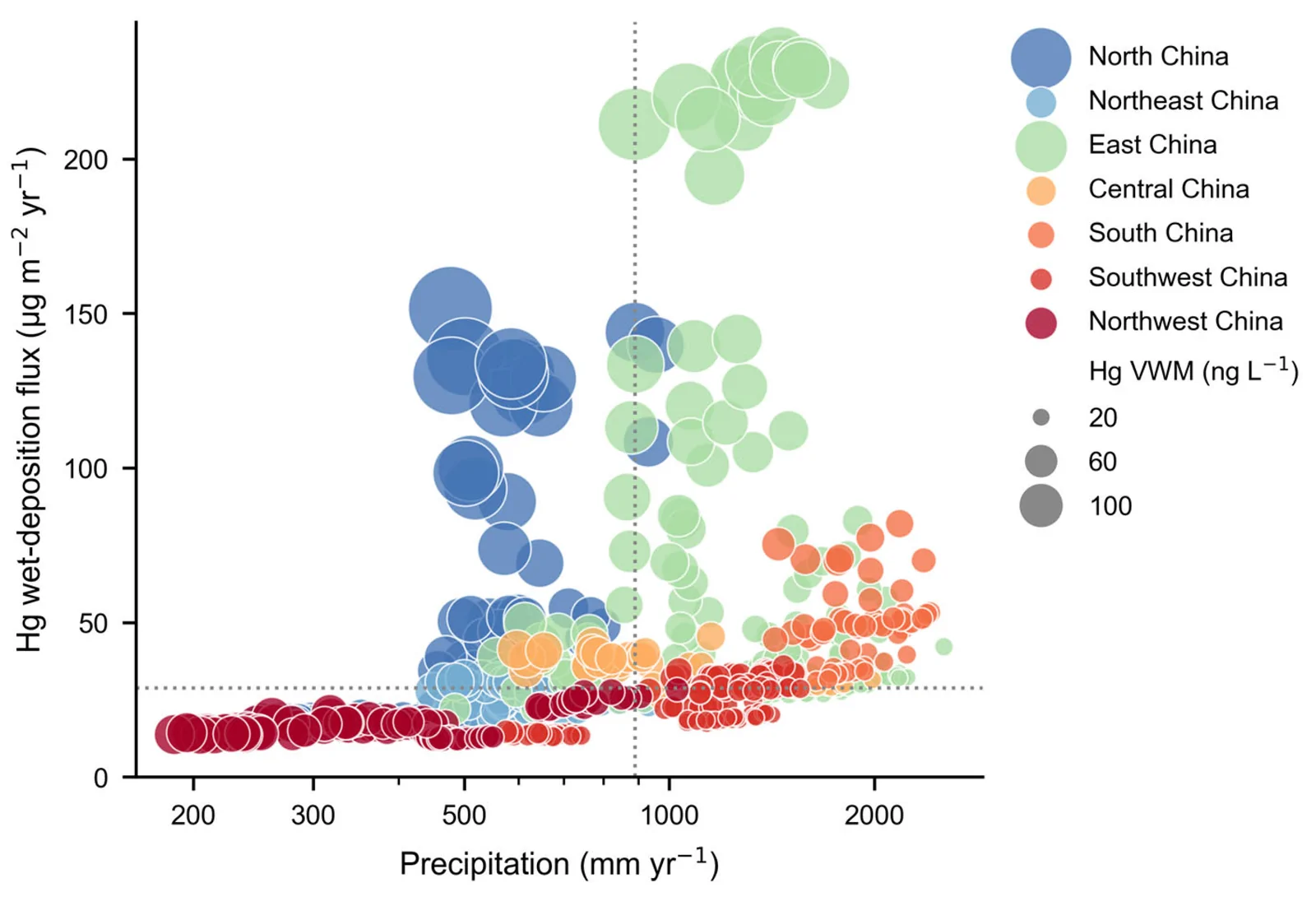
Relationship among annual precipitation, modeled wet deposition flux of Hg, and derived precipitation Hg VWM concentration across province-years. Bubble area represents VWM concentration; colors denote regions; gray dotted lines mark medians across all province-year points.

The joint distribution of annual precipitation, wet deposition flux, and precipitation concentration of Hg (Figure 6) illustrates the regional role of rainfall. Each bubble represents a province-year model estimate. Greater precipitation often coincided with greater flux, whereas concentration had no simple monotonic relationship with precipitation. South China and parts of the Western China Rain Zone had high precipitation and high flux with comparatively low concentrations [35,41]. Northwest China had low precipitation and low flux with relatively high concentrations; event-scale observations would be needed to test the suggested role of episodic removal [42]. East China had moderate-to-high rainfall with high flux and concentration. Regional studies and model intercomparisons likewise show that precipitation amount and seasonal structure are important for interpreting wet deposition in East and Southeast Asia [56,57].

Therefore, changes in precipitation Hg concentration do not necessarily match changes in deposited mass. Wet years may deliver large ecosystem inputs, especially in southern high-rainfall regions, even when concentration is relatively low. Conversely, dry regions may have higher concentrations but modest annual fluxes. Evaluations of atmospheric signals and ecological loading should report both metrics with the accompanying precipitation regime.

### 3.3. Predictor Importance and Associations

**Figure 7:**
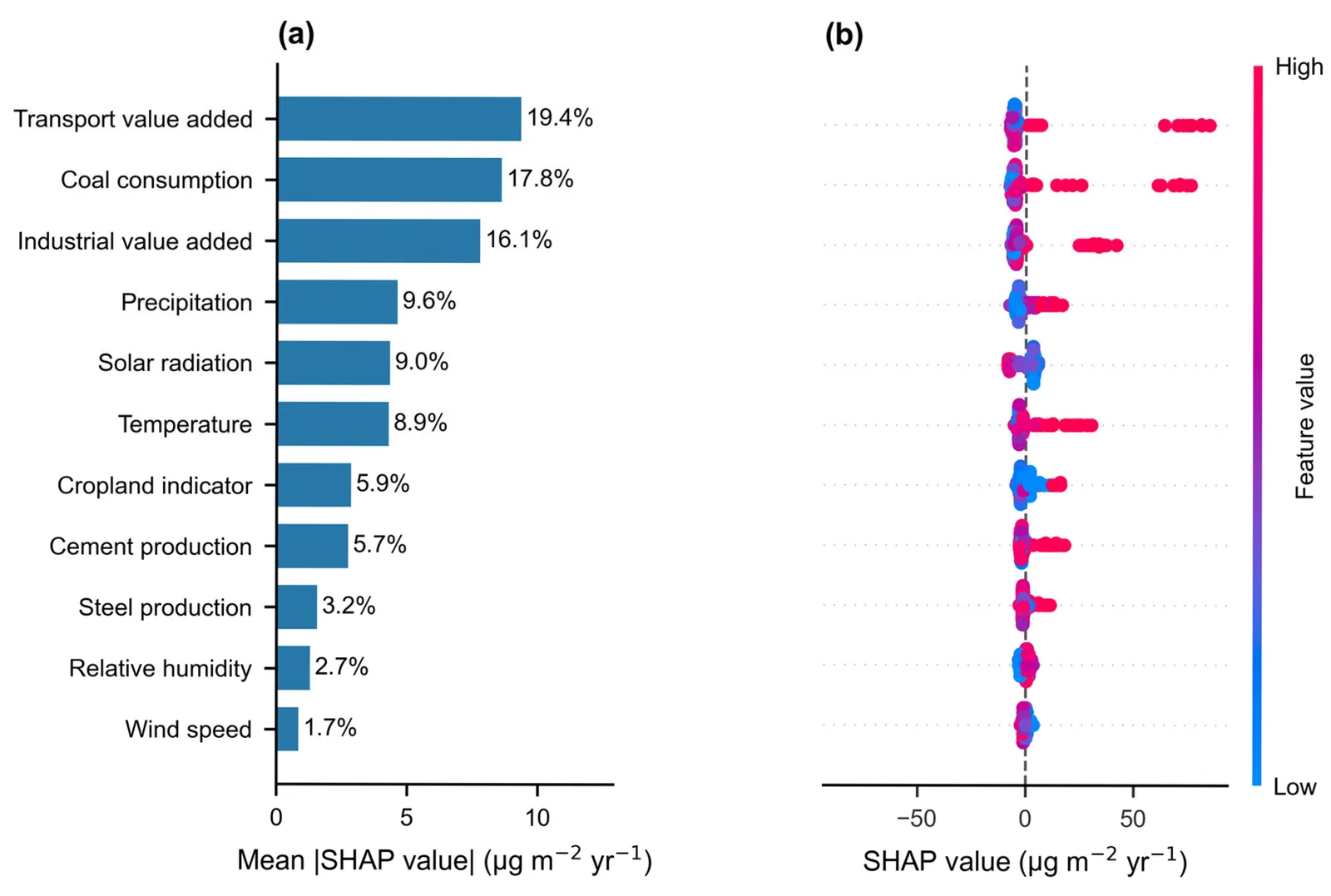
SHapley Additive exPlanations (SHAP) for the random forest model fitted to observations of wet deposition of Hg. (**a**) Predictors ranked by normalized mean absolute SHAP value; percentages sum to 100%. (**b**) Observation-level SHAP values, colored by relative predictor value. SHAP values describe associations within fitted model and do not establish independent causal effects.

Random forest modeling with SHAP interpretation identified the predictors most strongly associated with reconstructed wet deposition flux of Hg (Figure 7). Anthropogenic activity proxies and precipitation were prominent in the fitted model. These rankings describe the fitted reconstruction and should not be read as independent causal or emission source effects; Spearman correlations among predictors are shown in Figure S3.

Transport sector value-added (19.4%), coal consumption (17.8%), industrial value-added (16.1%), and precipitation (9.6%) together accounted for 62.9% of the normalized mean absolute SHAP importance. Solar radiation (9.0%) and air temperature (8.9%) followed, with smaller contributions from the cropland indicator (5.9%), cement production (5.7%), steel production (3.2%), relative humidity (2.7%), and wind speed (1.7%). High coal consumption and industrial activity were generally associated with higher modeled flux, consistent with the inventories and source-apportionment studies that identify combustion, metallurgy, industrial boilers, cement production, and transported emissions as important Hg sources [16,17,18,19,20,21,22,23,24,25,26,27,28]. Transport sector-value added is not a direct measure of Hg emissions. Although motor vehicles can emit Hg through fuel combustion [58], the variable used here is an economic activity indicator and is therefore interpreted primarily as a proxy for correlated urban economic activity, fuel consumption, and energy demand rather than as a source-specific Hg emission inventory.

Meteorological associations were nonlinear. Precipitation was the highest-ranked meteorological predictor by mean absolute SHAP value; higher values generally increased modeled flux, consistent with its role in wet removal [5,6,7,32,35,37,56]. Removing precipitation from the predictor set changed the site-grouped RMSE from 47.8 to 47.3 µg m^−2^ yr^−1^ (Figure S1). Higher air temperature was also associated with positive SHAP values, but this could represent covariance with warm-season oxidation, transport, or rainfall rather than an isolated temperature effect. High solar radiation tended to have negative or near-zero associations, potentially because it covaried with drier conditions; photochemical reduction of oxidized Hg may also have contributed [8,9]. Relative humidity and wind speed had weaker marginal associations at the annual national scale.

The cropland indicator had a modest positive association with modeled flux but should not be interpreted as a direct source effect. It could integrate covariance with population, energy use, land disturbance, vegetation, or soil–atmosphere exchange. Hg(0) exchange over natural surfaces is regulated by radiation, temperature, soil moisture, and disturbance [10,11], while watershed and wetland studies connect atmospheric deposition with runoff export and downstream loading [39,40]. Biomonitoring of contaminated landscapes shows that ecological signals reflect both source strength and surface retention [13].

Several limitations qualify the interpretation of Figure 7 and the national reconstruction. The literature sample is geographically and temporally uneven, with sampling intervals of uneven days and incomplete flux-source metadata. Seven Chinese sites were observed in a coordinated field study [35], and 17 sites contributed to a global wet deposition network analysis [5]; the 120 coordinate sites compiled here cover a wider area but are much less standardized. Site grouping reduced repeated-site leakage, yet leave-one-region-out performance was weaker, and removing high-flux observations reduced *R*^2^. Annual predictors, correlated activity proxies, the carried-forward coal input, and a cropland indicator with national high values further constrained interpretation. The maps describe exploratory, literature-constrained estimates, not verified local hotspots. Denser standardized monitoring and complete observation provenance are needed before local extremes or policy effects can be assessed reliably.

## 4. Conclusions

By integrating observations from 58 published studies with meteorological, land surface, and socioeconomic predictors, this study produced a broad-scale reconstruction of wet deposition of atmospheric Hg across China for 2000–2022. The fitted model used observations at 120 coordinate sites. Site-grouped validation achieved a pooled *R*^2^ = 0.596 and an RMSE = 47.80 µg m^−2^ yr^−1^, but leave-one-region-out performance was weaker. The reconstructed national annual mean flux ranged from 20.06 to 26.19 µg m^−2^ yr^−1^, while the derived precipitation Hg VWM concentration ranged from 28.02 to 37.12 ng L^−1^ and reached its highest value in 2022. The two metrics did not evolve synchronously because precipitation modifies mass transfer to the surface.

The regional patterns reinforced this distinction. Southern high-rainfall regions had comparatively low precipitation Hg concentrations and high fluxes, whereas drier northwestern regions had relatively high concentrations but low total flux. Transport sector value-added, coal consumption, industrial value-added, and precipitation ranked highest in SHAP importance, but these results represent model associations among correlated proxies rather than causal source attribution. The reconstruction can support cautious comparison of broad patterns; it cannot replace site-level monitoring or identify local exposure hotspots. For ecological assessment, precipitation Hg concentration, deposition flux, and rainfall regime should be reported together. Wet deposition quantifies an ecosystem input, not direct human dose; future work should link denser monitoring with retention, methylation, food web transfer, and population contact.

## Data Availability

The raw data supporting the conclusions of this article will be made available by the authors on request.

## Supplementary Materials

The following supporting information can be downloaded at: https://www.mdpi.com/article/10.3390/toxics14100879/s1, Data S1: Compiled dataset of atmospheric Hg wet-deposition observations in China used in this study; Table S1: Predictors used in the fitted model; Table S2: Validation results; Figure S1: RMSE from site-grouped five-fold validation after predictor or record restrictions; Figure S2: National maps of log10-transformed modeled wet deposition flux of Hg in 2000, 2010, and 2022; Figure S3: Spearman rank correlations among the 11 predictors across the fitted observations.
