# Supplementary material for: Spatiotemporal Variability in and Factors Associated with Wet Deposition of Atmospheric Mercury in China from 2000 to 2022
Source: Toxics. 2026 Sep 30;14(10):879. doi: 10.3390/toxics14100879 (PMC13709951; doi:10.3390/toxics14100879)
Supplement: Supplementary file 1 [file toxics-14-00879-s001.zip › Supplementary Information.pdf]

Supplementary Information

Spatiotemporal Variability in and Factors Associated with Wet Deposition of Atmospheric Mercury in China from 2000 to 2022

Xi Cheng [1,*], Zhihao Zeng [1], Jiaqi Wu [1], Chenxi Zhao [1], Shuhua Zeng [1] and Lei Liu [2,3,*]

[1] School of Chemical and Environmental Engineering, China University of Mining and Technology—Beijing, Beijing 100083, China; 2310340229@student.cumtb.edu.cn (Z.Z.); 2310340225@student.cumtb.edu.cn (J.W.); 2310340311@student.cumtb.edu.cn (C.Z.); 2310340310@student.cumtb.edu.cn (S.Z.)

[2] State Key Laboratory of Severe Weather Meteorological Science and Technology, Chinese Academy of Meteorological Sciences, Beijing 100081, China

[3] Key Laboratory of Atmospheric Chemistry of CMA, Chinese Academy of Meteorological Sciences, Beijing 100081, China

* Correspondence: xicheng@cumtb.edu.cn (X.C.); leiliu@cma.gov.cn (L.L.)

Table S1. Predictors used in the fitted model.

Predictor	Source	Native resolution	Transformation
Precipitation	CMFD 2.0	3-hourly; 0.1 degree	No
Near-surface air temperature	CMFD 2.0	3-hourly; 0.1 degree	No
Relative humidity	CMFD 2.0; derived field in supplied data	3-hourly; 0.1 degree	No
10-m wind speed	CMFD 2.0	3-hourly; 0.1 degree	No
Downward shortwave radiation	CMFD 2.0	3-hourly; 0.1 degree	No
Cropland indicator	GLC_FCS30 land-cover family	30 m source aggregated to 0.1 degree	log1p
Coal consumption	National Bureau of Statistics of China	Provincial annual	log1p
Cement production	National Bureau of Statistics of China	Provincial annual	log1p
Steel production	National Bureau of Statistics of China	Provincial annual	log1p
Industrial value added	National Bureau of Statistics of China	Provincial annual	log1p
Transport-sector value added	National Bureau of Statistics of China	Provincial annual	log1p

Table S2. Validation results. Grouped and regional results use pooled out-of-fold predictions; the random-split row gives the median across 50 test splits. Errors are in μg m^{-2} yr^{-1}.

Validation method	R^2	RMSE	MAE
grouped 5-fold by site	0.596	47.80	29.40
stratified grouped 5-fold by site (flux quartiles)	0.574	49.11	29.03
leave-one-region-out stress test	0.224	66.29	38.33
repeated random 75/25 split (50 repeats)	0.503	46.74	29.40

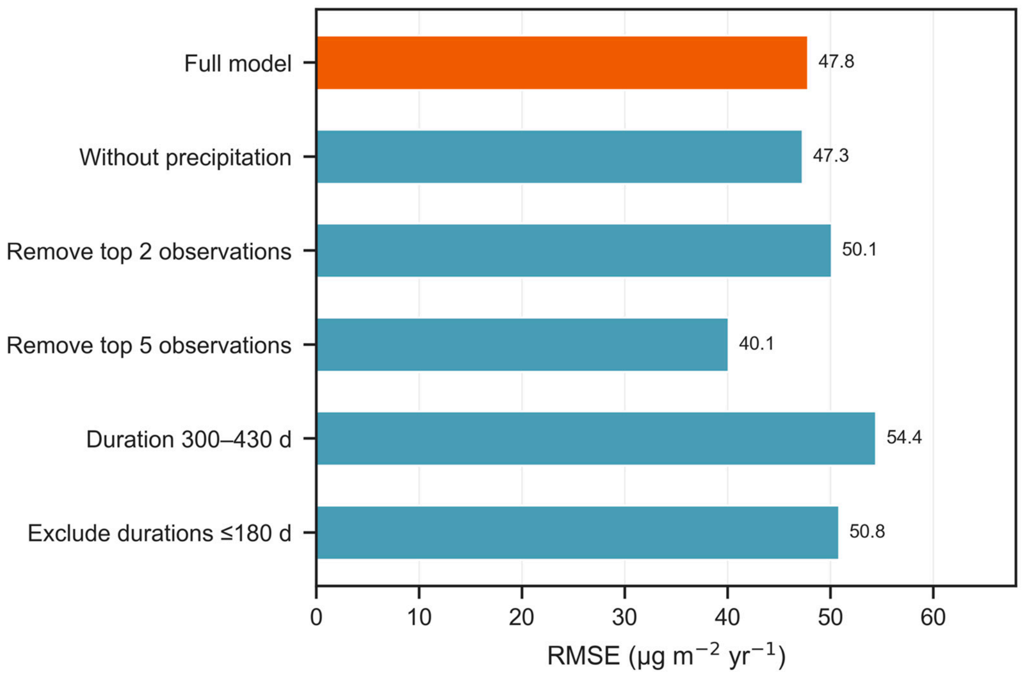

Figure S1. RMSE from site-grouped five-fold validation after predictor or record restrictions. The full model, no-precipitation model, removal of the two or five highest observations, 300–430-day subset, and exclusion of intervals of 180 days or less are shown.

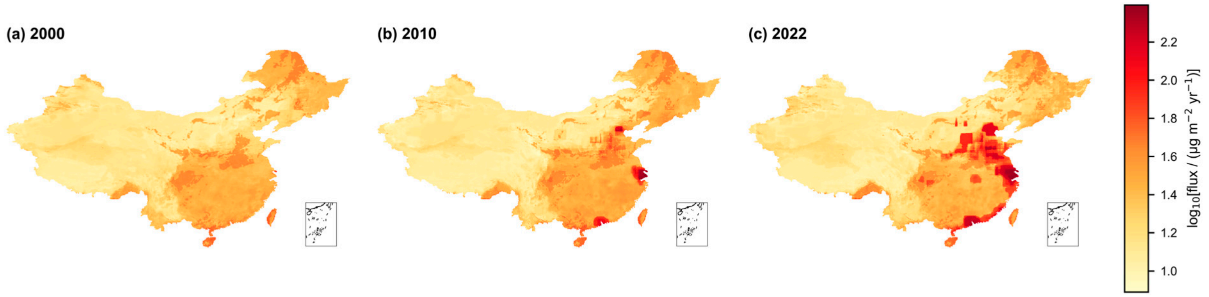

Figure S2. National maps of log10-transformed modeled Hg wet-deposition flux in 2000, 2010, and 2022. The three panels share one color scale.

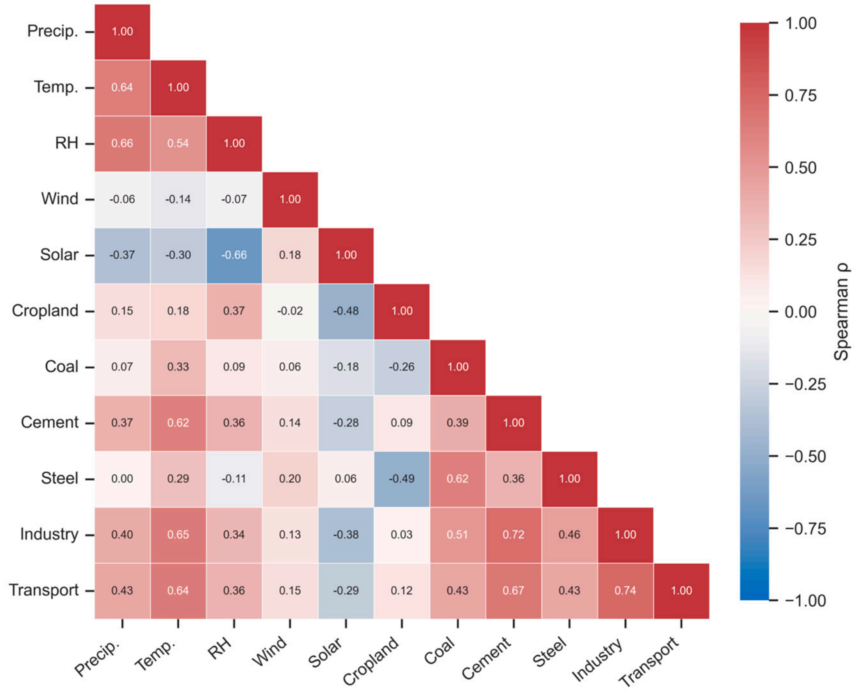

Figure S3. Spearman rank correlations among the 11 predictors across the fitted observations.
